# Role of maternal health and infant inflammation in nutritional and neurodevelopmental outcomes of two-year-old Bangladeshi children

**DOI:** 10.1371/journal.pntd.0006363

**Published:** 2018-05-29

**Authors:** Jeffrey R. Donowitz, Heather Cook, Masud Alam, Fahmida Tofail, Mamun Kabir, E. Ross Colgate, Marya P. Carmolli, Beth D. Kirkpatrick, Charles A. Nelson, Jennie Z. Ma, Rashidul Haque, William A. Petri

**Affiliations:** 1 Division of Infectious Diseases and International Health, University of Virginia, Charlottesville, Virginia, United States of America; 2 Division of Pediatric Infectious Diseases, Children’s Hospital of Richmond at Virginia Commonwealth University, Richmond, Virginia, United States of America; 3 Department of Statistics, University of Virginia, Charlottesville, Virginia, United States of America; 4 Division of Parasitology, International Centre for Diarrhoeal Disease Research, Bangladesh, (icddr,b), Dhaka, Bangladesh; 5 Child Development Unit, International Centre for Diarrhoeal Disease Research, Bangladesh, (icddr,b), Dhaka, Bangladesh; 6 Department of Medicine, University of Vermont College of Medicine, Burlington, Vermont, United States of America; 7 Division of Developmental Medicine, Boston Children’s Hospital, Harvard Medical School, Boston, Massachusetts, United States of America; 8 Harvard Graduate School of Education, Boston, Massachusetts, United States of America; 9 Department of Public Health Sciences, University of Virginia, Charlottesville, Virginia, United States of America; The Hospital for Sick Children, CANADA

## Abstract

**Background:**

Previous studies have shown maternal, inflammatory, and socioeconomic variables to be associated with growth and neurodevelopment in children from low-income countries. However, these outcomes are multifactorial and work describing which predictors most strongly influence them is lacking.

**Methodology/Principal findings:**

We conducted a longitudinal study of Bangladeshi children from birth to two years to assess oral vaccine efficacy. Variables pertaining to maternal and perinatal health, socioeconomic status, early childhood enteric and systemic inflammation, and anthropometry were collected. Bayley-III neurodevelopmental assessment was conducted at two years. As a secondary analysis, we employed hierarchical cluster and random forests techniques to identify and rank which variables predicted growth and neurodevelopment. Cluster analysis demonstrated three distinct groups of predictors. Mother’s weight and length-for-age Z score (LAZ) at enrollment were the strongest predictors of LAZ at two years. Cognitive score on Bayley-III was strongly predicted by weight-for-age (WAZ) at enrollment, income, and LAZ at enrollment. Top predictors of language included Rotavirus vaccination, plasma IL 5, sCD14, TNFα, mother’s weight, and male gender. Motor function was best predicted by fecal calprotectin, WAZ at enrollment, fecal neopterin, and plasma CRP index. The strongest predictors for social-emotional score included plasma sCD14, income, WAZ at enrollment, and LAZ at enrollment. Based on the random forests’ predictions, the estimated percentage of variation explained was 35.4% for LAZ at two years, 34.3% for ΔLAZ, 42.7% for cognitive score, 28.1% for language, 40.8% for motor, and 37.9% for social-emotional score.

**Conclusions/Significance:**

Birth anthropometry and maternal weight were strong predictors of growth while enteric and systemic inflammation had stronger associations with neurodevelopment. Birth anthropometry was a powerful predictor for all outcomes. These data suggest that further study of stunting in low-income settings should include variables relating to maternal and prenatal health, while investigations focusing on neurodevelopmental outcomes should additionally target causes of systemic and enteric inflammation.

## Author summary

Children from low-income settings experience linear growth faltering and neurodevelopmental delay that have been associated with maternal, socioeconomic, and infectious/inflammatory variables. Given the interdependent nature of these associations, understanding which variables are the best predictors of poor outcomes has been difficult. We conducted a longitudinal study of Bangladeshi children from birth to two years and collected predictors assessing maternal, inflammatory, and socioeconomic aspects of early childhood in Bangladeshi children. We conducted a random forests analysis to rank predictors associated with growth and neurodevelopment. Linear growth was best predicted by birth anthropometry and maternal weight. Cognitive function was predicted by birth anthropometry, socioeconomic status, and systemic inflammation. The receipt of the rotavirus vaccine and a combination of systemic inflammatory, maternal, and socioeconomic variables predicted language score. Motor score was predicted by systemic inflammation with enteric inflammatory markers having a reverse relationship. Social-emotional development was predicted by systemic inflammation, birth anthropometry, and economic means. This work demonstrates that specific pathways are responsible for different aspects of growth and development. Our data suggest that studies investigating pediatric stunting in low-income settings should focus on maternal and prenatal variables while those focused on neurodevelopmental outcomes should additionally target causes of systemic and enteric inflammation.

## Introduction

159 million children under five years old, or 23.8% of the world’s population in this age range, are stunted (length-for-age Z score [LAZ] < -2 standard deviations [SD]) [1]. In a pooled analysis, stunting conferred a hazard ratio of 2.28 for mortality prior to five years of age with severe stunting (LAZ < -3 SD) having a hazard ratio of 5.48 [2]. It has also been suggested that growth deficits early in life lead to obesity, type II diabetes, and metabolic disturbances later in life [3]. Height has been positively associated with earnings suggesting that early life adversity affecting growth leads to an immense loss of “human capital” throughout much of the world [4]. Additionally, stunting and infection have been associated with neurodevelopmental deficits, compounding the loss to productivity [3].

Both growth and neurodevelopment are multifactorial, which makes designing effective interventions difficult, especially in low- and middle- income countries (LMICs) where a variety of interconnected insults are present. Systemic inflammation, febrile episodes, lack of primary vaccines, lower socioeconomic status, and poor sanitation have all been directly associated with stunting in children from LMICs [5–11]. Environmental enteric dysfunction (EED), hallmarked by enteric inflammation, has also been shown to be associated with deficits in linear growth [6, 12–14]. Importantly, EED has been identified as a distinct entity from diarrheal disease although pathogen carriage may play a role in its development [15–18].

In addition to postnatal factors such as nutrition and inflammation, prenatal and maternal factors have also been correlated with growth. Maternal anthropometrics and maternal education have both been associated with stunting in children living in LMICs [7, 9, 19, 20]. In a multinational study, a 1 cm increase in maternal height was associated with a 1.2% decrease in risk of child mortality [21]. Additionally, birth anthropometry is a strong predictor of postnatal growth suggesting prenatal insults effect stunting [6, 9].

Associations between aspects of childhood life in LMICs and neurodevelopmental outcomes have also been described. Lower neurodevelopmental scores have been associated with diarrheal disease in some studies [22–24] but not in others [25]. The effect of enteric infection on neurodevelopment may be pathogen-specific as deficits have been associated with giardiasis and cryptosporidiosis specifically [23, 26]. Early childhood systemic inflammation and febrile illness have also been associated with poor neurodevelopmental outcomes [27, 28]. On a population level, average national IQ was associated with overall burden of infectious diseases suggesting an inflammatory pathway mediating effects on neurodevelopment [29]. Additionally, stunting has been associated with poor neurodevelopmental outcomes although the nature of this relationship remains undefined [3, 30–33].

Different types of insults affect separate aspects of neurodevelopment [34]. Prenatal and maternal factors including maternal malnutrition have been associated with decreased problem solving and motor function [35, 36]. Early life anthropometrics have been associated with cognitive and language function [34]. In one study breastfeeding was associated with improved language skills but not social-emotional/behavioral skills [37]. Neonatal sepsis has been linked to decreased motor and cognitive function but not social-emotional function [38, 39]. Systemic inflammation in animal models and increased intestinal permeability in humans has been linked to social-emotional function [40, 41]. The combination of these findings suggest a need to assess neurodevelopment directly and with subscale analysis.

Many of the variables associated with poor growth and neurodevelopment are not independent but rather interdependent [42, 43]. This has made analysis of their individual importance in these outcomes difficult. Our objective was to clarify which aspects of childhood in LMICs are the strongest predictors of both growth and neurodevelopment.

## Methods

We conducted a longitudinal study from birth to two years in Bangladeshi infants and collected data assessing maternal health, socioeconomic status, sanitary conditions, and enteric and systemic inflammation in early childhood. We utilized random forests analysis, an ensemble machine learning method, to identify and rank predictors of linear growth and neurodevelopment. The predictability of top variables from random forests was estimated from a linear model and expressed as the percentage of variation. To validate our findings, the same set of predictive variables used for random forests were also analyzed in a penalized linear model with smoothly clipped absolute deviation (SCAD) penalty.

### Study design

The Performance of Rotavirus and Oral Polio Vaccines in Developing Countries (PROVIDE) study is a longitudinal study of Bangladeshi infants. The primary objective of the PROVIDE study was to determine if there was an association between EED and the underperformance of oral vaccines with the analysis described here being secondary. Detailed methods of this study have been published elsewhere [6, 44]. Briefly, 700 children were enrolled within one week of birth. Participants were randomized to receive the Rotarix oral rotavirus vaccine or not and all children received the oral polio vaccine. A rolling admission spanned from May 2011 through November 2014. Results of this study examining the association of biomarkers of EED and oral polio vaccine failure, rotavirus vaccine failure, growth through one year of age, small intestine bacterial overgrowth, the impact of enteropathogens on oral rotavirus and polio vaccination, and the association of Rotarix vaccination and serum zinc levels with severe rotavirus diarrhea have been published elsewhere [6, 45–47].

### Study population

PROVIDE was conducted in the urban borough of Mirpur in Dhaka, Bangladesh. The area is densely populated with a mean of 5 people living in 1.5 rooms. Over 95% of the construction is of tin or mud brick. Uncovered sewer drains flow throughout the area and abut 59% of dwellings. Our subjects tended to come from the lower socioeconomic strata of Mirpur due to the area in which recruitment occurred and the location of our study clinic.

### Rotavirus vaccination

All children received vaccines, administered by the study staff, included in the Bangladesh Expanded Programme on Immunization, including the oral polio vaccine. Children were randomized after enrollment to receive the oral rotavirus vaccine Rotarix (GlaxoSmithKline plc., Middlesex, UK) or not. Children randomized to the Rotarix vaccine arm received two doses at 10 and 17 weeks of age. Rotavirus vaccination was assessed as a dichotomous predictor, either receiving the vaccine or not.

### Biomarker, socioeconomic, anthropometric, and Bayley assessments

Stool and serum for biomarker analysis was collected within 7 days of the scheduled study visit and were immediately stored at 4°C. Samples were transported from our field office to The International Centre for Diarrhoeal Disease Research, Bangladesh Parasitology Laboratory and stored at -80 °C within 4 hours of collection. They were then pulled and analyzed in batches.

Plasma activin, plasma ferritin, plasma anti-lipopolysaccharide (LPS) antibody, plasma retinol binding protein (RBP), plasma soluble cluster designation 14 (sCD14), plasma zinc, fecal myeloperoxidase, fecal calprotectin, fecal alpha-1 antitrypsin, fecal neopterin, fecal Reg 1B, plasma vitamin D, plasma C-reactive protein (CRP), and Cytomegalovirus (CMV) status were assessed via commercially available ELISA kits. CMV status was dichotomized to positive or negative per manufacturer’s specifications (Abcam, inc. Cambridge, Ma USA). Mannitol recovery was assessed via urine high performance liquid chromatography after giving children a standardized mannitol load. Plasma cytokine analysis was conducted via a commercially available Human BioPlex Pro assay [6, 44].

Plasma activin, plasma cytokines, and plasma CRP were chosen as markers of systemic inflammation. Plasma ferritin, RBP, and zinc were chosen as both acute phase reactants and key nutritional variables. Vitamin D was also selected as a key nutritional measure. Fecal myeloperoxidase, fecal calprotectin, fecal alpha-1 antitrypsin, fecal neopterin, and fecal Reg 1B were selected as markers of enteric inflammation and damage. Mannitol recovery, anti-LPS Ab, and sCD14 were selected as markers of intestinal permeability.

Diarrheal surveillance to calculate days of diarrhea was conducted by field research assistants who visited the homes of the participants twice per week. Maternal and socioeconomic data were collected at the time of enrollment via questionnaire. Mother’s level of education achieved was collected then dichotomized to any formal education or no formal education. Presence of an open drain directly outside the home, family’s use of a septic tank or toilet (as opposed to slab latrines, pit latrines, open latrine, hanging latrines, or open defecation), use of a toilet shared by other families, and use of any method of improved water treatment were also assessed via questionnaire. Anthropometric assessment (including maternal anthropometry) was conducted by study physicians trained in the procedure using measuring boards, measuring tape, and calibrated scales as appropriate for size and age. Anthropometry was measured at enrollment and at 16 scheduled study visits throughout the 2-year study period.

A trained psychologist assessed neurodevelopmental scores at two years of age using a version of the Bayley Scales of Infant and Toddler Development, Third Edition (Bayley-III) that was adapted to be culturally appropriate to Bangladeshi children. Despite cultural adaptation, this version was not normalized to the Bangladeshi population. This version of the Bayley III has been used in other studies by our group and was shown to have high short term (within 7 days) retest reliability (r > 0.80) and high inter-observer reliability (r = 0.99) [27, 28].

### Statistical analysis

LAZ, weight-for-age Z score (WAZ), and weight-for-height Z score (WHZ) were calculated using the World Health Organization software WHO Anthro (version 3.2.2). CRP was measured at 4 time points (6, 18, 40, and 53 weeks). The variable “CRP index” was created as a measure of sustained inflammation. For each measurement, if a child was in the top 50^th^ percentile for that time point, they were given a score of 1. “CRP Index” was created by summing the scores given for all four CRP measurements and thus ranged from 0 to 4. Cytokines were discretized into <50^th^ percentile, the 50^th^– 75^th^ percentile, and >75^th^ percentile. All other variables were either dichotomous or continuous based on the nature of the variables.

Separate datasets were created for anthropometric and neurodevelopmental outcomes. Any child with an incomplete data set for the specified outcome was removed from the analysis. Outliers in predictors, defined as any value > 5 SD from the mean, were excluded from the analysis. 28 subjects for anthropometric analysis and 22 subjects for neurodevelopmental analysis were excluded. Outliers were not assessed in outcome measurements. Differences in enrollment characteristics between the remaining subjects and the original cohort who had complete enrollment data were assessed via Mann-Whitney U tests and χ^2^ tests as appropriate.

Pearson correlation for all predictive variables was calculated using the dataset constructed for anthropometry. Hierarchical clustering to examine relationships between variables was performed and depicted as a cluster dendrogram. A dissimilarity index of 1.75 was chosen for the clustering cutoff in order to describe how larger groups of the variables were related. Each variable was color-coded based on which of the three clusters it was in and this color-coding was used to identify variables in the random forests plots.

Outcomes of interest for the predictive models included LAZ at two years of age, the change in LAZ from enrollment to two years (ΔLAZ), and the four components of the Bayley-III (cognitive, language, motor, and social-emotional). A separate random forests analysis was conducted for each of our outcomes of interest to select and rank predictive variables. Conditional random forests analyses were performed to account for the correlations between predictors with a threshold of Pearson’s correlation coefficient ≥0.2. Variable importance values (VIMP) were calculated for all predictors and then scaled based on the predictor with the highest VIMP in that analysis (sVIMP). In order to determine the direction of the association between variables and outcomes, dependence plots between predictors and outcomes of interest were generated for the top 15 predictors.

As a validation, penalized linear regression analyses with SCAD penalty were performed on the same datasets. For SCAD analyses, dummy variables for cytokine measurements were created and if either the 50^th^– 75^th^ or the >75^th^ percentile was selected, the other was forced into the model.

To assess the predictability from the random forests analyses, for each outcome, a mean squared error was calculated using the predicted value from the random forests model and the observed values, and then percentage of variation explained by the predictors was calculated. Percentage of variation explained was also calculated from the SCAD model. All analyses were done using R software. The statistical package ‘party’ version 1.2–2 from February 27, 2017 was used for conditional random forests. The statistical package ‘grpreg’ version 3.0–2 from July 11, 2016 was used for variable selection with SCAD.

### Ethics statement

The PROVIDE study was approved by the Research Review and Ethics Review Committees at The International Centre for Diarrhoeal Disease Research, Bangladesh and by the Institutional Review Boards at the University of Virginia and the University of Vermont. Informed consent was obtained from parents for their child’s participation in this study. All data analyzed were anonymized.

## Results

**Fig 1:**
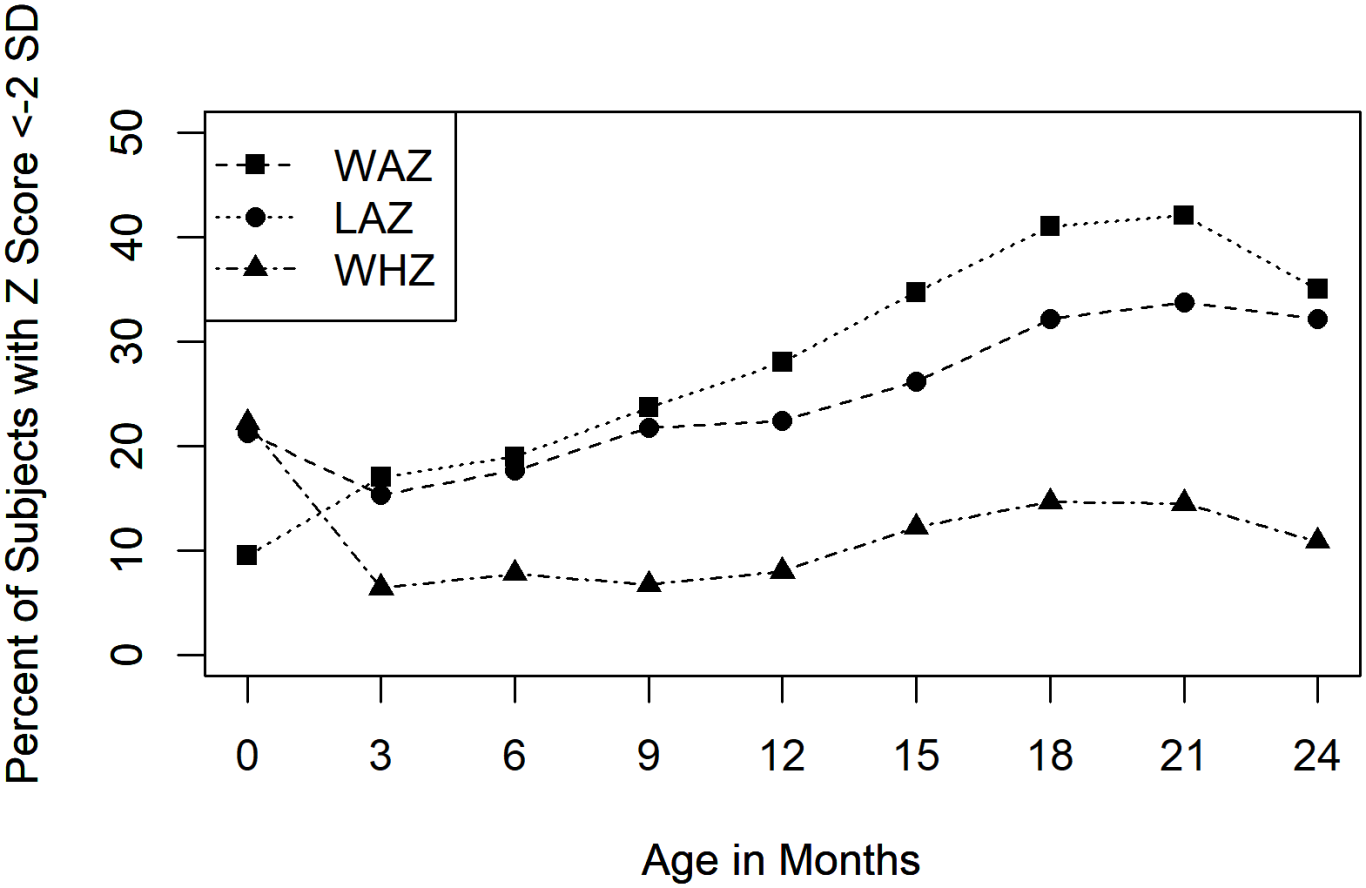
Anthropometry over the first 2 years of life for the 371 children with complete anthropometric measurements. Height and weight were taken at 3-month intervals and transformed to standardized Z scores. The X-axis depicts the child’s age while the Y-axis depicts the percentage of children in the cohort who had a LAZ, WAZ, or WHZ <-2 SD.

Complete data sets of predictors included in this study were available for 371 subjects for anthropometry and 308 subjects for Bayley-III neurodevelopmental assessment once outliers were removed (Table 1). 661 subjects had complete enrollment datasets. There was no significant difference in enrollment characteristics between either the anthropometry dataset or the neurodevelopmental dataset used in this analysis and the original cohort except for maternal education (Table 2). The average LAZ at two years was -1.7±1.6 SD. The average ΔLAZ from enrollment to two years was -0.9±1.6 SD. 33.3% of children had an LAZ <-2 SD by two years of age (Fig 1). The average Bayley-III scores were 90.7±5.8, 98.6±8.5, 94.9±7.4, and 91.2±5.8 for cognitive, language, motor, and social-emotional, respectively.

**Table 1:** Variables measured in the PROVIDE study utilized in random forests models to predict linear growth and Bayley-III neurodevelopmental scores at two years of age.

At Enrollment	Week 6	Week 12	Week 18	Week 24	Other
- Expenditure	-Activin	-Alpha-1 antitrypsin	-Anti-LPS Ab	-Mannitol Recovery	-CMV IgG positivity at week 40
-Gender	-Anti-LPS Ab	-Calprotectin	-Days of Diarrhea		-Elevated CRP index (measured at 6, 18, 40, 53 weeks)
-Income	-Ferritin	-Mannitol Recovery	-Days of exclusive breastfeeding through 18 weeks		-Rotarix vaccination (given at 10 and 17 weeks)
-LAZ	-RBP	-Myeloperoxidase	-Ferritin		
-Maternal Education	-Reg 1b	-Neopterin	-IL-10		
-Mother’s Height at enrollment	-sCD14	-Reg 1b	-IL-1b		
-Mother’s Weight at enrollment	-Vitamin D		-IL-4		
-Open drain outside the home	-Zinc		-IL-5		
-Septic Tank/Toilet			-IL-6		
-Shared Toilet			-IL-7		
-Water treatment			-MIP1b		
-WAZ			-RBP		
-WHZ			-sCD14		
			-TNFα		
			-Vitamin D		
			-Zinc		

**Table 2:** Enrollment characteristics of children who completed 2 year anthropomorphic assessments compared with those who did not.

Variable	Children with Incomplete Data for Anthropometry at 2 years (n = 290)	Children with Complete Data for Anthropometry at 2 years (n = 371)	p value
WAZ at Enrollment	-1.3 ± 0.8	-1.3 ± 0.8	0.31
LAZ at Enrollment	-0.9 ± 0.9	-0.9 ± 0.4	0.80
WHZ at Enrollment	-1.3 ± 0.9	-1.3 ± 0.9	0.14
Age at Enrollment (days)	4.8 ± 1.7	5.1 ± 1.6	0.09
Vaccinated with Rotarix	131 (45.2)	193 (52.0)	0.08
Any Maternal Education	223 (76.9)	347 (66.6)	0.004
Expenditure (taka)*	11,607 ± 7833	11,529 ± 6,742	0.63
Income (taka)*	13,104 ± 10,981	12,621 ± 8,093	0.64
Mother’s Weight at Enrollment (kg)	49.4 ± 9.7	49.2 ± 9.2	0.91
Mother’s Height at Enrollment (cm)	150.3 ± 5.7	150.4 ± 5.4	0.61
Male	164 (56.6)	188 (51.7)	0.13
Treated Water	170 (58.6)	233 (62.8)	0.27
Septic Tank/Toilet	147 (50.7)	197 (53.1)	0.54
No Shared Toilet	247 (85.2)	317 (85.4)	0.99
No Open Drain Outside Home	118 (40.7)	149 (40.2)	0.89

Data are expressed in mean ± SD for continuous measures and count (%) for discrete measures. P values calculated by Mann-Whitney U test for continuous variables and χ^2^ test for discrete variables.

* 1 USD = approximately 80.5 Taka

### Hierarchical clustering

The hierarchical cluster analysis demonstrated three distinct major clusters, similar to previous analysis of this data [6] (S1 Fig). Systemic cytokines continued to cluster tightly as in the previous analysis. However, enrollment anthropometry, as opposed to week 18 anthropometry used previously, more closely correlated with sanitation. Economic status (income and expenditure) closely clustered with biomarkers of enteric inflammation. CRP index was also in this cluster. Overall, variables from each cluster tended to represent that cluster across our random forests analyses.

### Predictors identified by random forests analysis

**Fig 2:**
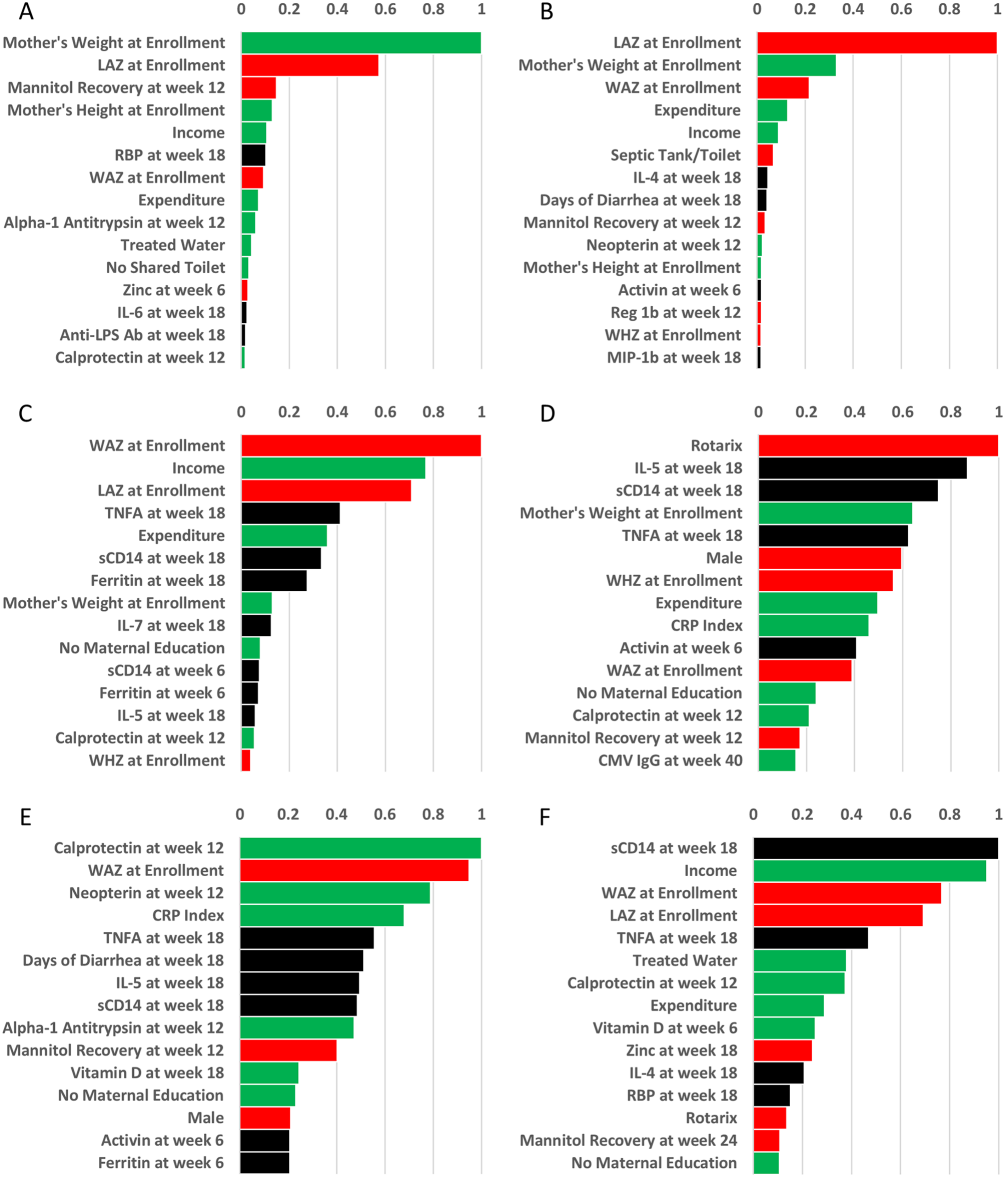
Random forests plots of the top 15 variables selected for linear growth and performance on the Bayley-III scales of infant and Toddler Development. Random forests models were constructed for LAZ at two years (A), the change in LAZ from enrollment to two years (B), and Bayley-III scores at two years of age. The Bayley-III is comprised of 4 components, namely cognitive (C), language (D), motor (E), and social-emotional (F). Color-coding correlates to the three clusters identified when all variables were analyzed using Pearson’s correlation (S1 Fig).

sVIMP values from the conditional random forests analyses are depicted for the top ranked variables in Fig 2 and dependence plots for all outcomes in S2–S7 Figs. For LAZ at two years as a static measure, maternal weight (index sVIMP, 1.0) and LAZ at enrollment (sVIMP 0.57) were substantially stronger predictors than the remainder. There was a substantial drop in sVIMP between LAZ at enrollment and the next highest predictor, which was mannitol recovery at week 12 (sVIMP 0.15). For ΔLAZ from enrollment to two years, LAZ at enrollment became the strongest predictor (index sVIMP, 1.0), followed by maternal weight (sVIMP 0.33). Overall, birth anthropometry and maternal weight far surpassed all other variables in terms of their ability to predict anthropometry at two years and growth (Fig 2A & 2B).

In analysis of Bayley-III outcomes, in general, inflammation was of greater importance. For cognitive score WAZ at enrollment was the top predictor (index sVIMP, 1.0), followed by income (sVIMP 0.77), and LAZ at enrollment (sVIMP 0.71). Inflammatory variables such as TNFα at 18 weeks (sVIMP 0.41), sCD14 at week 18 (sVIMP 0.33), and ferritin at week 18 (sVIMP 0.27), as well as the economic variable expenditure (sVIMP 0.36) were weaker predictors (Fig 2C).

Language scores were predicted by presence/absence of the rotavirus vaccine (index sVIMP, 1.0), IL 5 at week 18 (sVIMP 0.87), sCD14 at week 18 (sVIMP 0.75), maternal weight (sVIMP 0.64), TNFα at week 18 (sVIMP 0.63), male gender (sVIMP 0.60), and WHZ at enrollment (sVIMP 0.56). Language scores were influenced by a diverse combination of factors ranging across all three groups of our cluster analysis (Fig 2D).

For motor function, predictors included calprotectin at 12 weeks (index sVIMP, 1.0) followed by WAZ at enrollment (sVIMP 0.95), neopterin at week 12 (sVIMP 0.79), CRP index (sVIMP 0.68), TNFα at week 18 (sVIMP 0.56), days of diarrhea at week 18 (sVIMP 0.51), IL 5 at week 18 (sVIMP 0.50), sCD14 at week 18 (sVIMP 0.49), alpha-1 antitrypsin at week 12 (sVIMP 0.47), and mannitol recovery at week 12 (sVIMP 0.40). Motor score was strongly predicted by variables that grouped tightly in our hierarchical cluster analysis and included biomarkers of enteric inflammation. Biomarkers of systemic inflammation and birth anthropometry were also strong predictors (Fig 2E).

The highest-ranking predictor of social-emotional function was sCD14 (index sVIMP, 1.0) followed by income (sVIMP 0.95), WAZ at enrollment (sVIMP 0.77), LAZ at enrollment (sVIMP 0.69), and TNFα at week 18 (sVIMP 0.47). Social-emotional score appeared to be influenced by gut barrier integrity, economic means, systemic inflammation, and birth anthropometrics (Fig 2F).

The estimates of the percentage of variance explained in our outcomes at two years of age from the conditional random forests analyses were 35.4% and 34.3% for LAZ and ΔLAZ respectively; 42.7% for cognitive score, 28.1% language score, 40.8% for motor score, and 37.9% for social-emotional score.

### Predictors identified by variable selection using smoothly clipped absolute deviation (SCAD)

In order to identify risk factors that independently predicted outcomes, variable selection utilizing SCAD was done. Overall, SCAD selected 18 of 23 (78%) of predictors that random forests assigned sVIMP values >0.50. For anthropometry alone, SCAD selected 3 of 3 (100%) of predictors with sVIMP values >0.50. This included LAZ at enrollment for both LAZ at two years and ΔLAZ from enrollment to two years. For LAZ at two years it also included mother’s weight.

For Bayley-III outcomes alone, SCAD selected 15 of 20 (75%) of variables with random forests sVIMP values >0.50. For the cognitive component both WAZ at enrollment and income were selected while LAZ at enrollment was not. For language, only WHZ at enrollment was not selected. SCAD confirmed the importance of rotavirus vaccine status, IL 5, sCD14, mother’s weight, TNFα, and male gender in predicting language scores. SCAD analysis of Bayley-III motor scores selected calprotectin, WAZ at enrollment, neopterin, TNFα, and IL 5, all of which random forests selected with sVIMP >0.50. However SCAD failed to select CRP index and days of diarrhea at week 18. For social-emotional predictors, SCAD overlapped with random forests on 3 of 4 variables with sVIMP >0.50 including sCD14, income, and WAZ at enrollment. LAZ at enrollment was not selected (Table 3).

**Table 3:** Variables selected by both random forest (top 10 selected variables) and SCAD (all selected variables) analyses for the outcomes of linear growth and performance on the Bayley III scales of infant and Toddler Development.

Random Forests*		SCAD	
Selected Variable	sVIMP	Selected Variable	Direction of Association
**LAZ at week 104**			
Mother’s Weight	1.00	Mother’s weight	(+)
LAZ at Enrollment	0.57	LAZ at Enrollment	(+)
Mannitol Recovery at week 12	0.15	Expenditure	(+)
Mother’s Height	0.13	Alpha-1 Antitrypsin at week 12	(+)
Income	0.11	IL 4 at week 18, >75%ile	(+)
RBP at week 18	0.10	IL 4 at week 18, 50–75%ile	(+)
WAZ at Enrollment	0.09	IL 10 at week 18, >75%ile	(+)
Expenditure	0.07	IL 10 at week 18, 50–75%ile	(+)
Alpha-1 Antitrypsin at week 12	0.06	Calprotectin at week 12	(+)
Treated Water	0.04		
**ΔLAZ from Enrollment to week 104**			
LAZ at Enrollment	1.00	LAZ at Enrollment	(-)
Mother’s Weight	0.33	Mother’s weight	(+)
WAZ at Enrollment	0.22	Expenditure	(+)
Expenditure	0.13	IL 4 at week 18, >75%ile	(+)
Income	0.09	IL 4 at week 18, 50–75%ile	(+)
Septic Tank/Toilet	0.07	Calprotectin at week 12	(+)
IL 4 at week 18	0.05	Alpha-1 Antitrypsin at week 12	(+)
Days of Diarrhea at week 18	0.04		
Mannitol Recovery at week 12	0.03		
Neopterin at week 12	0.02		
**Bayley III–Cognitive Score**			
WAZ at Enrollment	1.00	WAZ at Enrollment	(+)
Income	0.77	Income	(+)
LAZ at Enrollment	0.71	TNFα at week 18, >75%ile	(-)
TNFα at week 18	0.41	TNFα at week 18, 50–75%ile	(+)
Expenditure	0.36	sCD14_at week 18	(-)
sCD14 at week 18	0.33	Ferritin at week 18	(+)
Ferritin at week 18	0.27	IL 7 at week 18, >75%ile	(-)
Mother’s Weight	0.13	IL 7 at week 18, 50–75%ile	(-)
IL 7 at week 18	0.13	No Maternal Education	(-)
No Maternal Education	0.08	Anti-LPS Ab at week 18	(-)
		Anti-LPS Ab at week 6	(-)
		Male	(-)
		Calprotectin at week 12	(-)
		Neopterin at week 12	(+)
**Bayley III–Language Score**			
Rotarix	1.00	Rotarix	(+)
IL 5 at week 18	0.87	IL 5 at week 18, >75%ile	(+)
sCD14 at week 18	0.75	IL 5 at week 18, 50–75%ile	(+)
Mother’s Weight	0.64	sCD14 at week 18	(-)
TNFα at week 18	0.63	Mother’s weight	(+)
Male	0.60	TNFα at week 18, >75%ile	(-)
WHZ at Enrollment	0.56	TNFα at week 18, 50–75%ile	(+)
Expenditure	0.50	Male	(-)
CRP Index	0.46	No Maternal education	(-)
Activin at week 6	0.41	WAZ at Enrollment	(+)
		Income	(+)
		Calprotectin at week 12	(-)
		MPO at week 12	(-)
		Ferritin at week 6	(+)
**Bayley III–Motor Score**			
Calprotectin at week 12	1.00	Calprotectin at week 12	(-)
WAZ at Enrollment	0.95	WAZ at Enrollment	(+)
Neopterin at week 12	0.79	Neopterin at week 12	(+)
CRP Index	0.68	TNFα at week 18, >75%ile	(-)
TNFα at week 18	0.56	TNFα at week 18, 50–75%ile	(+)
Days of Diarrhea at week 18	0.51	IL 5 at week 18, >75%ile	(-)
IL 5 at week 18	0.50	IL 5 at week 18, 50–75%ile	(+)
sCD14 at week 18	0.49	sCD14 at week 18	(-)
Alpha-1 Antitrypsin at week 12	0.47	No Maternal education	(-)
Mannitol Recovery at week 12	0.40	Income	(+)
		Reg1B at week 6	(-)
		Anti-LPS Ab at week 18	(-)
		MPO at week 12	(+)
		Vit D at week 6	(+)
		Mother’s weight	(+)
		Ferritin at week 18	(+)
		IL 7 at week 18, >75%ile	(-)
		IL 7 at week 18, 50–75%ile	(-)
**Bayley III–Socioemotional Score**			
sCD14 at week 18	1.00	sCD14 at week 18	(-)
Income	0.95	Income	(+)
WAZ at Enrollment	0.77	WAZ at Enrollment	(+)
LAZ at Enrollment	0.69	TNFα at week 18, >75%ile	(-)
TNFα at week 18	0.47	TNFα at week 18, 50–75%ile	(+)
Treated Water	0.38	Treated Water	(+)
Calprotectin at week 12	0.37	Calprotectin at week 12	(-)
Expenditure	0.29	Vit D at week 6	(+)
Vitamin D at week 6	0.25	Zinc at week 6	(-)
Zinc at week 18	0.24	Mother’s height	(+)
		No Maternal education	(-)
		Male	(-)
		RBP at week 18	(-)
		Mannitol Recovery at week 12	(-)

*Only the top 10 variables shown

Linear regression models created by SCAD for each outcome produced estimates of variability for each outcome that were 18.0% for LAZ at two years, 17.9% for ΔLAZ, 17.5% for cognitive score, 12.9% for language score, 13.6% for motor score, and 17.6% for social-emotional score.

## Discussion

The key discovery of this work was ranking the importance of putative predictors of infant growth and neurodevelopment and demonstrating that they were different. LAZ at two years of age was predicted predominantly by maternal and birth anthropometrics. In contrast developmental scores were most prominently predicted by inflammatory biomarkers. These data suggest that interventions aimed to improve growth and neurodevelopment need to be directed at both improvements in maternal and neonatal nutrition and reduction of gut and systemic inflammation.

The finding that perinatal child and maternal anthropometry predicted linear growth reaffirms several studies showing birth anthropometrics are strong predictors of ΔLAZ, suggesting that catch up growth in children born small for gestational age or with intrauterine growth restriction is insufficient [48–51]. Additionally, our findings support previous work showing maternal anthropometry to influence infant growth [21, 50]. Fecal calprotectin and alpha-1 antitrypsin were positively associated with growth. Both markers can be elevated due to intestinal inflammation but have also been shown to be increased in breastfeeding children and thus may be a surrogate marker of improved nutrition in our analysis [52, 53]. However, our data showed that markers of systemic or enteric inflammation were not the strongest predictors of poor growth although studies have repeatedly shown an association [3, 15, 16, 54–59]. As random forests ranks predictors in order of their importance, it may be that the association between inflammation and growth noted in other studies is valid but that inflammation is not as important a driver of growth when compared with maternal or prenatal factors. Our results suggest that future investigation into the complex pathogenesis of growth stunting should include study of the prenatal period.

Our analysis of Bayley-III outcomes demonstrates differences in cognitive, language, motor, and social-emotional development pathways and suggests that different insults may influence separate aspects of neurodevelopment. Cognitive development was strongly affected by perinatal anthropometrics and economic variables although systemic inflammation also played a role. Although there is literature suggesting birth LAZ is predictive of cognitive outcomes [60], several studies have shown associations between LAZ as a static measure at other ages and cognitive function [31, 61–64]. However, these studies did not examine birth anthropometry as a confounder. Our results suggest that birth anthropometry may influence both future LAZ/growth and cognitive performance. Work showing that nutritional supplementation in early childhood had minimal or no effect on cognition support a maternal, prenatal, or non-nutritional (i.e. possibly inflammatory) cause of cognitive deficits [65–67]. However, a recent meta-analysis showed certain nutrients given postnatally including iron can affect cognitive development. Maternal nutritional supplementation in the first trimester was also associated with improved cognition [68]. The presence of ferritin and birth anthropometry as important predictors of cognitive function in our analysis supports these findings. Our analysis is consistent with previous work by our group that suggests systemic inflammation negatively effects cognitive development and work by others showing infectious diseases in early childhood were associated with lower cognitive function [28, 30, 61, 69].

Language scores in our analysis were predicted in part by vaccination against rotavirus, systemic inflammation, mother’s weight, and gender. In this study, the vaccine was shown to have an efficacy of 73.5% against severe rotavirus diarrhea [70]. As only days of diarrhea until 18 weeks was entered into our models, it may be that rotavirus vaccination was a marker of decreased diarrhea over a longer period, which contributed to improved language ability, possibly through a decrease in systemic inflammation. Studies of meningitis in children have repeatedly shown sensorineural hearing loss leading to language deficits to be associated with inflammation in the central nervous system [71]. As we did not measure hearing in our cohort, it is uncertain if the association of systemic inflammation and language deficits has a similar pathogenesis in children from LMICs. Our finding that male gender was associated with decreased language function is consistent with a large body of literature showing females to progress faster in language development [72, 73]. However, Rotavirus vaccination, maternal weight, and markers of systemic inflammation were all stronger predictors than gender. This would suggest that in addition to the direct effects of Rotavirus vaccination on diarrheal disease, downstream effects on development may be an additional benefit of adding the Rotavirus vaccine to national campaigns.

Motor score was associated with markers of systemic immune activation including TNFα, ferritin, CRP, and sCD14. Additionally calprotectin and neopterin were strongly associated with motor function and SCAD revealed a direct relationship. While these markers of enteric inflammation have been associated with poor linear growth in other studies, it may be that their anti-inflammatory effects are significant enough to limit a systemic effect of enteric inflammation and thus are neuroprotective. Anthropometry in older children has been associated with poor motor function but, again, our work would suggest birth anthropometrics to be a confounder in these analyses (45,48).

Social-emotional function was predicted by a diverse set of variables spanning all three groups in our cluster analysis with sCD14, income, and birth anthropometrics being the highest ranking. Mannitol recovery and fecal calprotectin were also negatively associated with social-emotional scores. Inflammation and specifically enteric inflammation has been associated with poor socio-emotional function in other settings including in studies of attention-deficit-hyperactivity-disorder and autism [41, 74–77]. Zinc levels were negatively associated with social-emotional score, which was a surprising and unexplained finding.

Our study reaffirms the findings from the first year analysis of this data that our measured predictors cluster into three distinct groups [6]. While systemic inflammation still clustered tightly, CRP index was more closely correlated with maternal, socioeconomic, and enteric inflammatory variables. This variation is likely due to use of the CRP index in our analyses instead of weeks 6 and 18 CRP values used in the previous work. Additionally, we used enrollment anthropometrics instead of week 18 anthropometrics. While week 18 values clustered with maternal anthropometrics, enrollment values clustered tightly with markers of sanitation [6]. This supports the findings from our random forests and SCAD analyses showing that maternal anthropometry is an important driver of postnatal growth. Birth anthropometrics appear more closely linked to risk factors with potential water, sanitation and hygiene (WASH) interventions. Given the prominence of birth anthropometry in all outcomes of interest in this study, future investigation is warranted to determine the effects of prenatal WASH interventions in expecting mothers, which may have high yield in mitigating the adverse effects of the LMIC environment on childhood growth and development.

Our study has several strengths. First was our relatively large sample size and the ability to collect multiple predictors related to complex biologic processes such as poverty, maternal health, enteric inflammation, and systemic inflammation. Additionally children were followed closely in semi-weekly household visits for two years to obtain neurodevelopmental and anthropometric data. Finally, we were able to utilize two distinct statistical methods, which had significant overlap in findings.

There are several procedural limitations that should be considered when examining this work. First, not all of the original 700 children in the cohort had all of the biomarkers measured. While comparison of enrollment characteristics showed no difference between the children included in the original cohort and those analyzed except for maternal education, the possibility remains of selection bias. Second, while our Bayley-III assessment was culturally adapted, it was not normalized to the Bangladeshi population. This limits our ability to compare the absolute values to an international population and define the extent of the neurodevelopmental delays documented by comparison. Third, information regarding the children’s home environment as it relates to home education and stimulation was not collected nor was information regarding dietary intake. These variables are known to affect scores on neurodevelopmental assessments and may represent unexamined confounders in our analysis [78]. Fourth, several variables collected including ferritin, RBP, and zinc may be difficult to interpret since they are both acute phase reactants and nutritional markers [79]. Finally, for biomarkers of inflammation other than CRP, a limited number of time points were sampled. This limits our ability to assess if we are measuring acute or chronic inflammation, which would improve our understanding of the inflammatory insult on our outcomes.

Previous work has shown that birth anthropometry, maternal education, infection, inflammation, and poverty can impact growth and neurodevelopment [27, 34, 60, 64, 80]. Our analyses suggest that there are several different pathways leading to poor linear growth and neurodevelopment which are likely interrelated. Given the prominence of maternal and prenatal factors in our analyses, future efforts to study linear growth and neurodevelopmental deficits in LMICs should include data collection on these variables. However, to fully assess factors affecting neurodevelopmental outcomes, postnatal effects including those from EED and infection will need to be considered as well.

## Supporting information

S1 Fig Cluster dendrogram of the variables collected in the PROVIDE study. All predictors entered into both random forests and SCAD analysis were compared using Pearson’s correlation. Three distinct clusters were noted (in Black, Green, and Red).
(TIF)

S2 Fig Dependence plots for the top 15 variables selected by random forests analysis for LAZ at week 104. Directionality of the relationship between outcome and predictor is depicted.
(TIF)

S3 Fig Dependence plots for the top 15 variables selected by random forests analysis for ΔLAZ from Enrollment to week 104. Directionality of the relationship between outcome and predictor is depicted.
(TIF)

S4 Fig Dependence plots for the top 15 variables selected by random forests analysis for Bayley-III—Cognitive Score. Directionality of the relationship between outcome and predictor is depicted.
(TIF)

S5 Fig Dependence plots for the top 15 variables selected by random forests analysis for Bayley-III—Language Score. Directionality of the relationship between outcome and predictor is depicted.
(TIF)

S6 Fig Dependence plots for the top 15 variables selected by random forests analysis for Bayley-III—Motor Score. Directionality of the relationship between outcome and predictor is depicted.
(TIF)

S7 Fig Dependence plots for the top 15 variables selected by random forests analysis for Bayley-III—Social-emotional Score. Directionality of the relationship between outcome and predictor is depicted.
(TIF)

S1 Checklist STROBE checklist: STROBE Checklist (https://www.strobe-statement.org/index.php?id=available-checklists) for cohort studies was completed. (PDF)

S1 Dataset Dataset used for analyses with Bayley-III scores as the outcomes. This file includes all predictive and Bayley-III outcome variables. It includes all children for whom we had a complete dataset, including Bayley-III scores, prior to outliers being removed.
(CSV)

S2 Dataset Dataset used for analyses with LAZ or ΔLAZ as the outcome. This file includes all predictive and anthropometric outcome variables. It includes all children for whom we had a complete dataset, including LAZ and ΔLAZ, prior to outliers being removed.
(CSV)

S3 Dataset Data Dictionary for S1 and S2 Datasets. This file contains a description of the variable codes used in the S1 and S2 Datasets as well as the units used for each variable.
(XLSX)
