# Supplementary figures and images for: Role of maternal health and infant inflammation in nutritional and neurodevelopmental outcomes of two-year-old Bangladeshi children
Source: PLoS Negl Trop Dis. 2018 May 29;12(5):e0006363. doi: 10.1371/journal.pntd.0006363 (PMC5993301; doi:10.1371/journal.pntd.0006363)

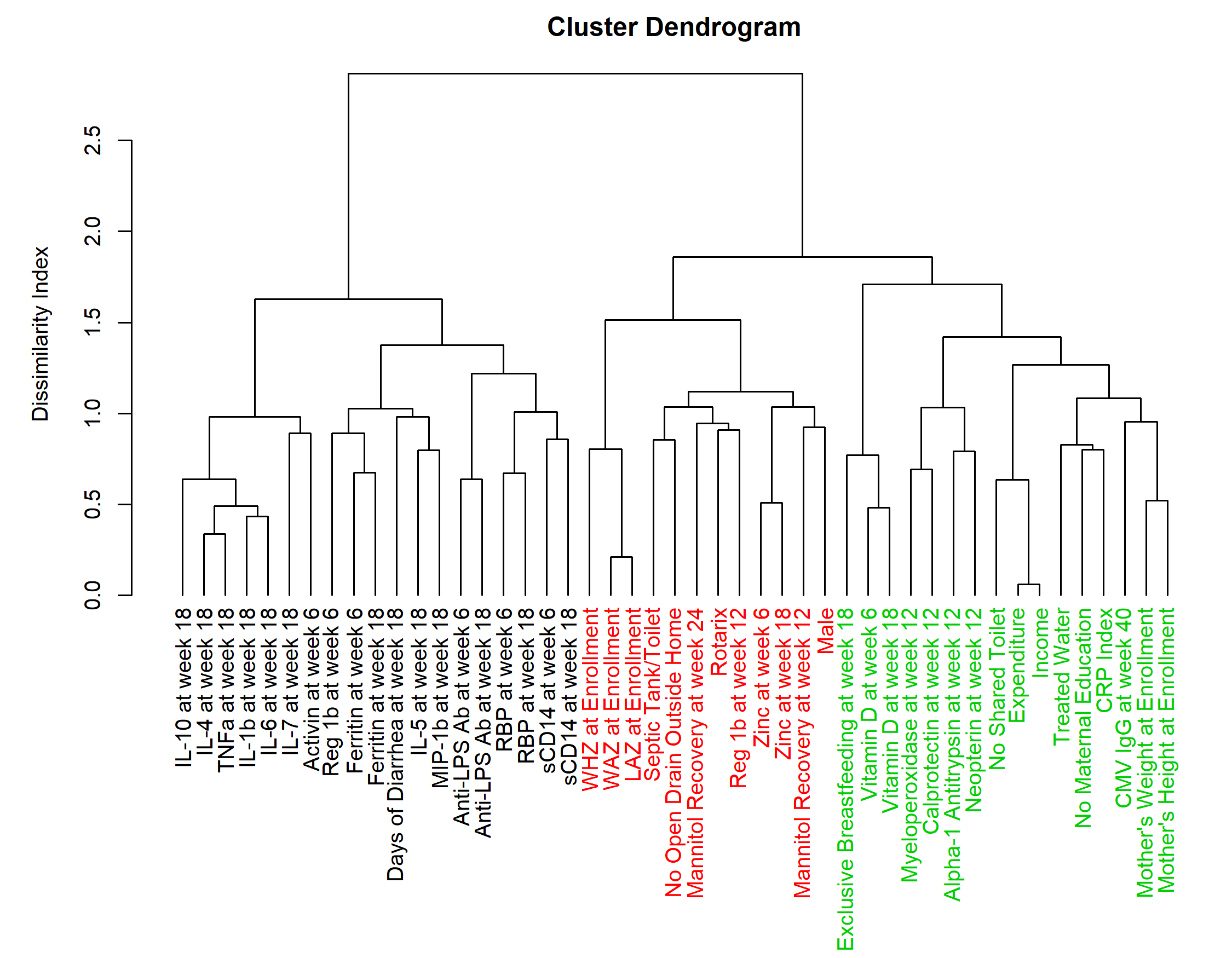

Supplement: S1 Fig — All predictors entered into both random forests and SCAD analysis were compared using Pearson’s correlation. Three distinct clusters were noted (in Black, Green, and Red). (TIF) [file pntd.0006363.s001.tif]

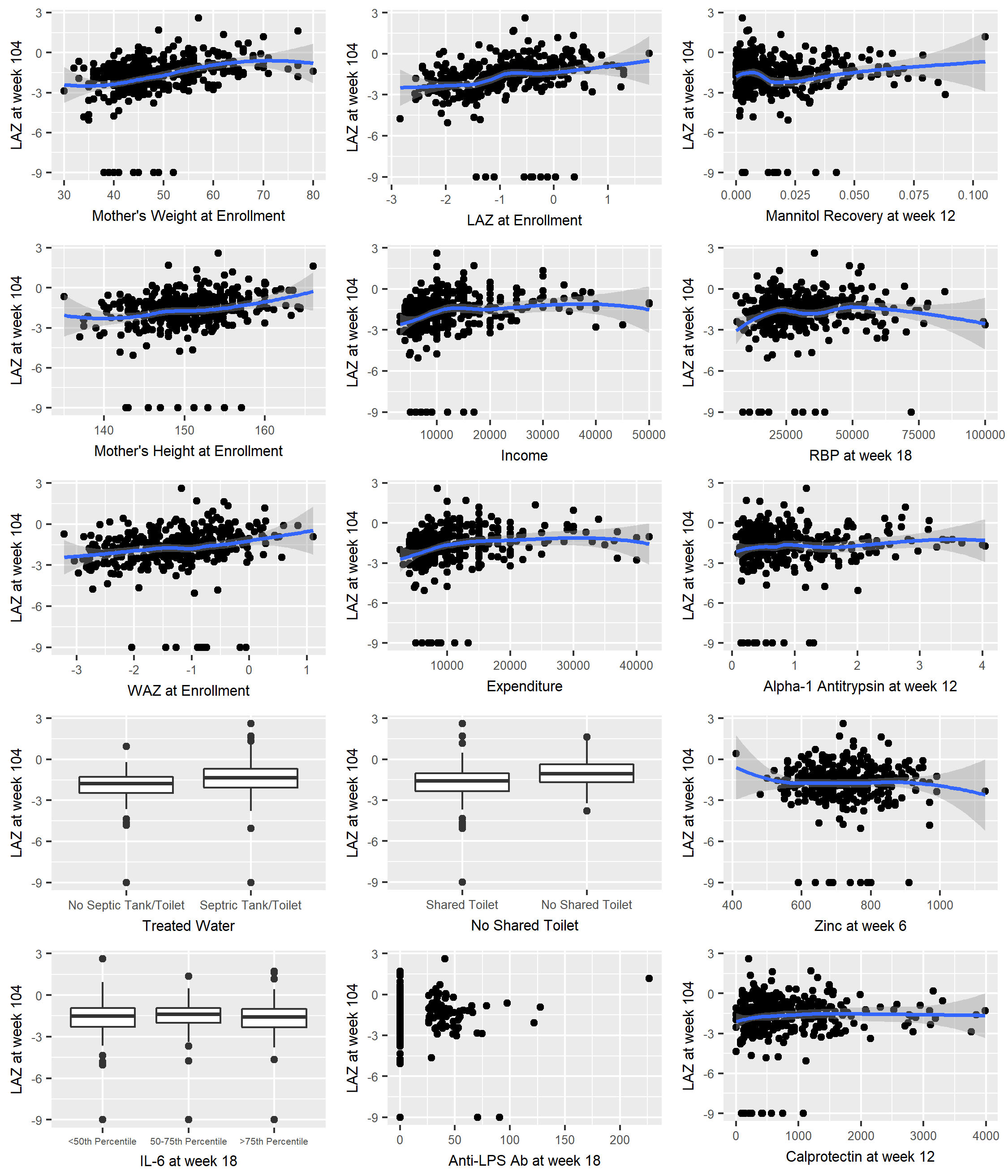

Supplement: S2 Fig — Directionality of the relationship between outcome and predictor is depicted. (TIF) [file pntd.0006363.s002.tif]

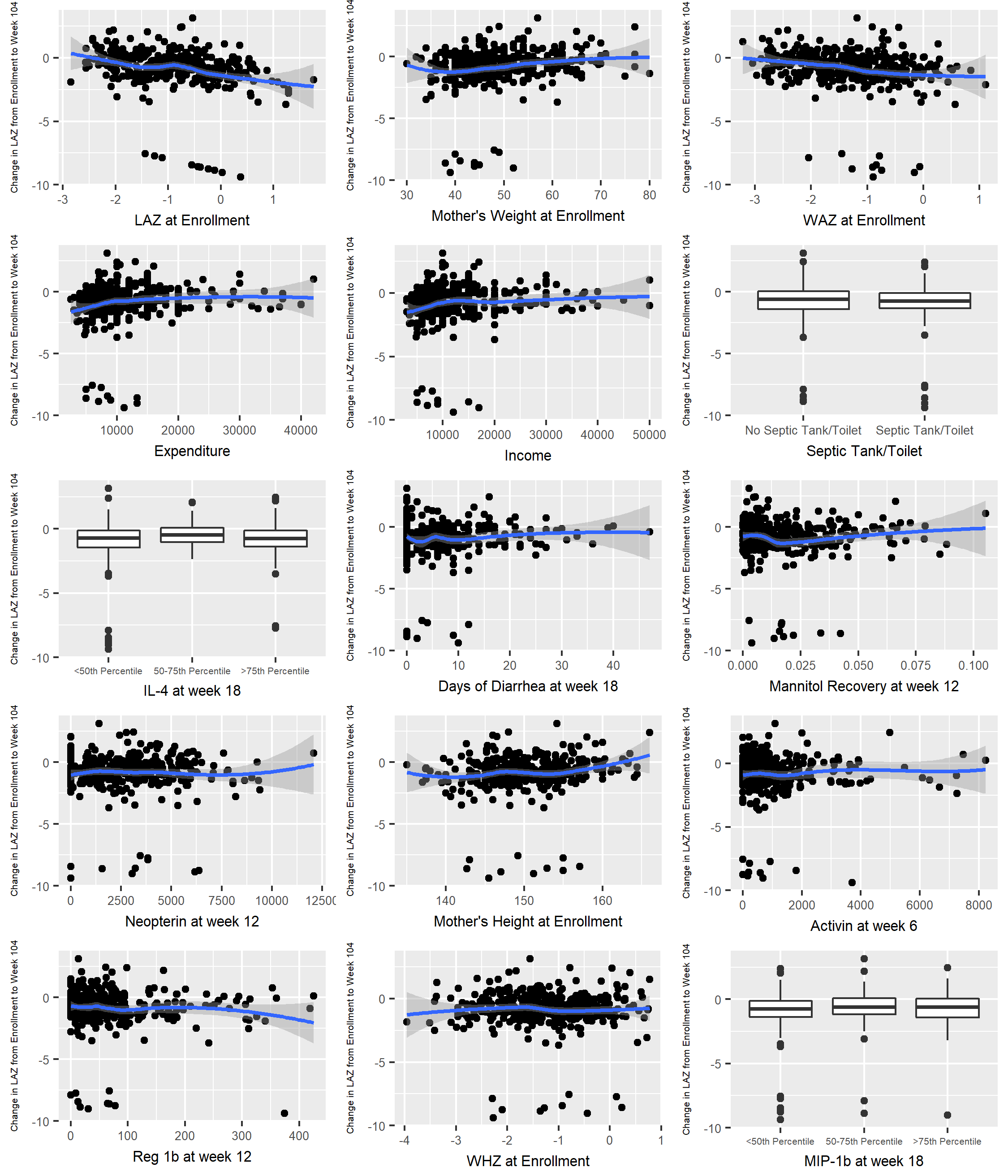

Supplement: S3 Fig — Directionality of the relationship between outcome and predictor is depicted. (TIF) [file pntd.0006363.s003.tif]

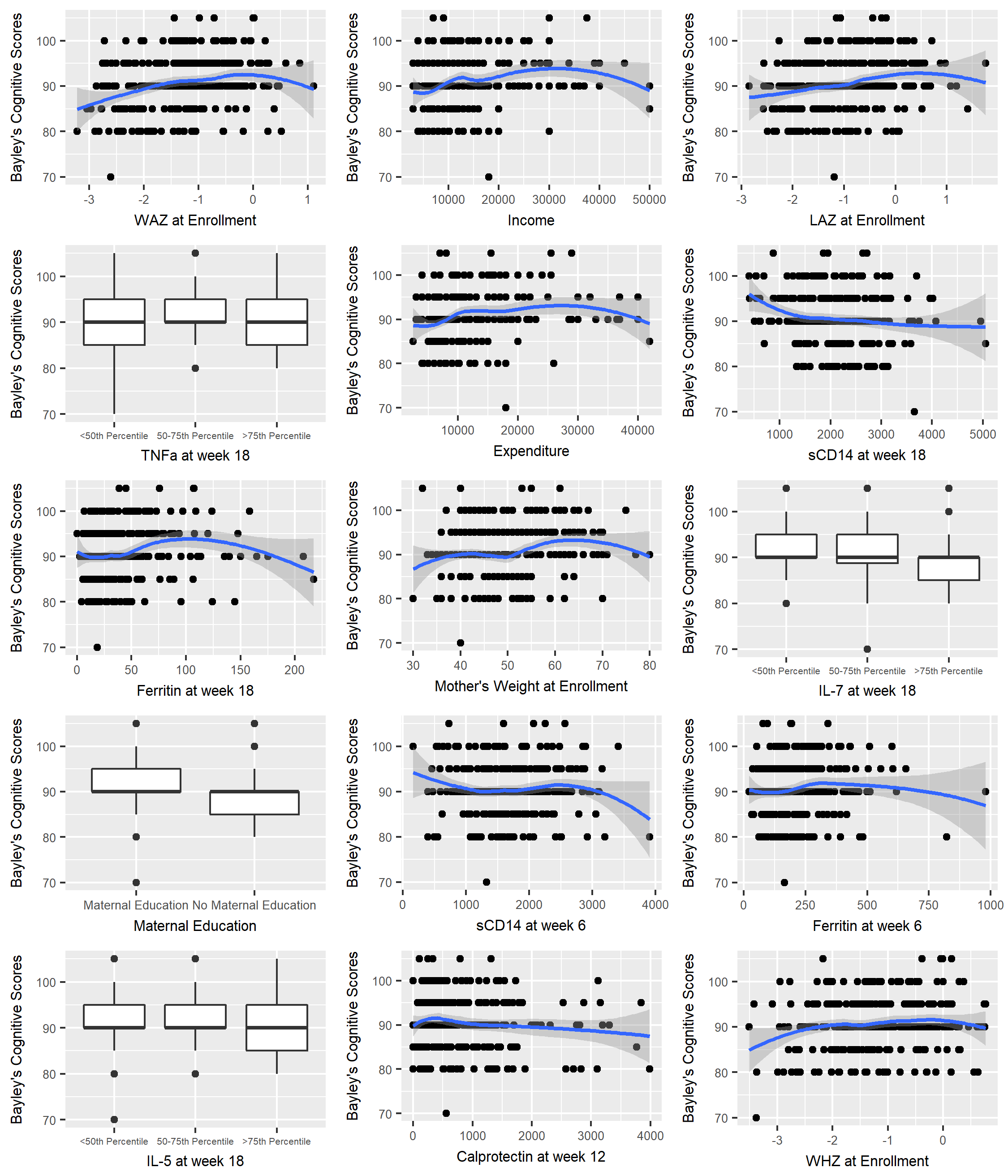

Supplement: S4 Fig — Directionality of the relationship between outcome and predictor is depicted. (TIF) [file pntd.0006363.s004.tif]

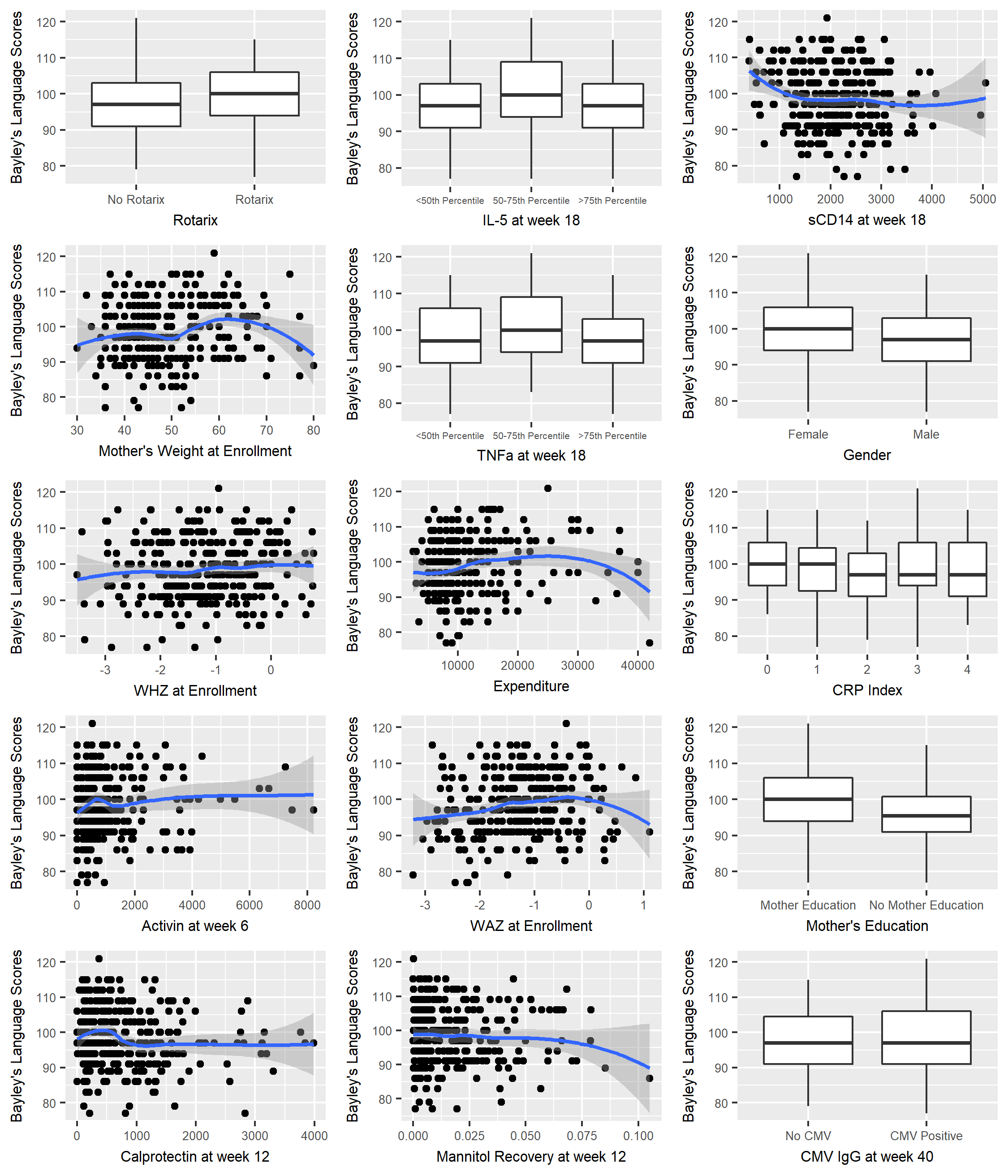

Supplement: S5 Fig — Directionality of the relationship between outcome and predictor is depicted. (TIF) [file pntd.0006363.s005.tif]

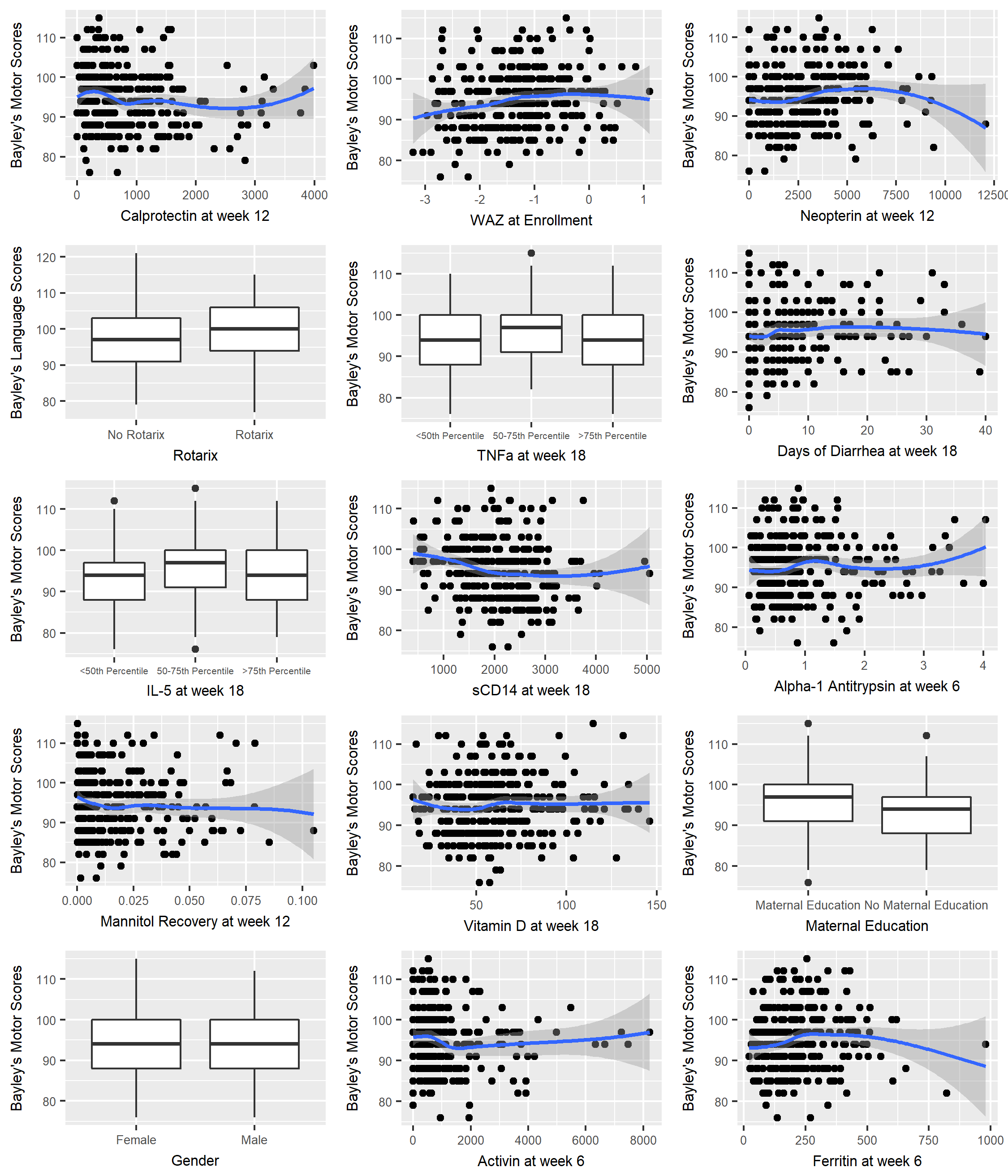

Supplement: S6 Fig — Directionality of the relationship between outcome and predictor is depicted. (TIF) [file pntd.0006363.s006.tif]

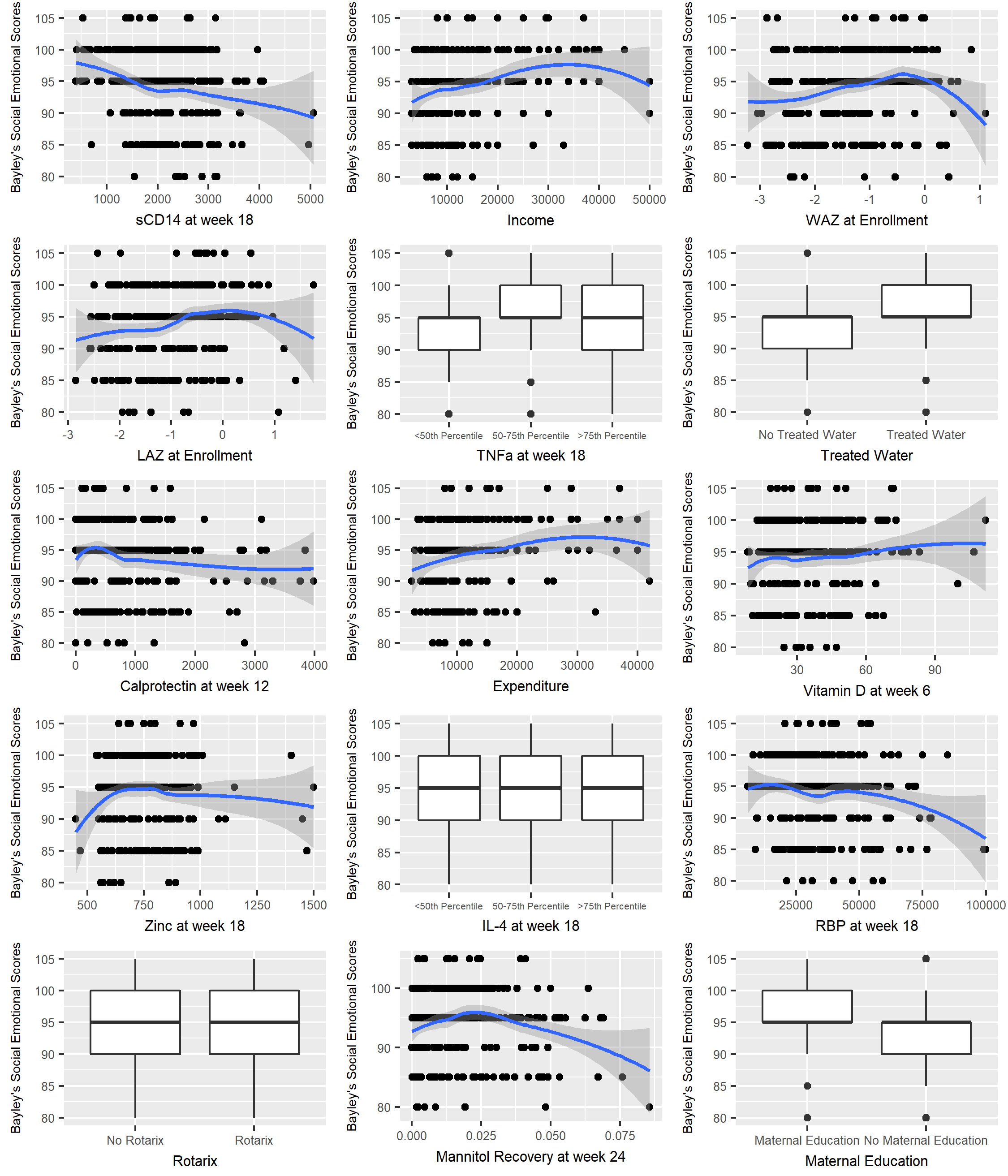

Supplement: S7 Fig — Directionality of the relationship between outcome and predictor is depicted. (TIF) [file pntd.0006363.s007.tif]
